# Radiological and Hematological Parameters Predicting Success of Medical Expulsive Therapy in Patients With Ureteral Calculus

**DOI:** 10.7759/cureus.67356

**Published:** 2024-08-21

**Authors:** Arshad Hasan, Vinod Kumar, Surya Kant Kumar, Mahesh M

**Affiliations:** 1 Department of Urology, Patna Medical College and Hospital, Patna, IND; 2 Department of Urology, All India Institute of Medical Sciences, Kalyani, IND; 3 Department of Urology, Narayan Medical College and Hospital, Rohtas, IND; 4 Department of Urology, Sri Devaraj Urs Medical College and R.L. Jalappa Hospital &amp; Research Centre, Kolar, IND

**Keywords:** hematological markers, radiological parameters, medical expulsive therapy, ureteral stones, urolithiasis

## Abstract

Background

Urolithiasis, characterized by the formation of stones in the urinary tract, is a common condition with significant morbidity. Medical expulsive therapy (MET) has emerged as a non-invasive treatment modality for facilitating the spontaneous passage of ureteral stones. However, MET success varies widely among individuals, and identifying predictive factors for treatment outcomes is crucial for optimizing patient care.

Objective

This study aimed to evaluate the predictive role of radiological and hematological parameters in the success of MET for ureteral stones.

Methods

A prospective observational cohort study was conducted at the Department of Urology, tertiary care center, Patna, involving consecutive patients who underwent MET for ureteral stones. Radiological parameters (stone size, location, and hydronephrosis) and hematological markers (C-reactive protein (CRP) and neutrophil-to-lymphocyte ratio (NLR)) were assessed as predictors of treatment success. Univariate and multivariate logistic regression analyses were performed to identify significant predictors of MET success.

Results

Among the 156 patients included in the study (mean age: 42 years; range: 18-50 years), radiological parameters, such as smaller stone size (<10 mm) and absence of hydronephrosis, were significantly associated with higher rates of MET success (p<0.05). Lower CRP levels (p<0.001) and NLR (p<0.05) were also predictive of treatment success. Logistic regression analysis confirmed the independent predictive value of these parameters after adjusting for potential confounders.

Conclusion

Radiological and hematological parameters are valuable predictors of the success of MET for ureteral stones. Incorporating these predictive factors into clinical decision-making can optimize treatment outcomes and minimize unnecessary interventions in patients with a higher likelihood of spontaneous stone passage.

## Introduction

Urolithiasis, commonly known as urinary tract stones or calculi, is a prevalent condition affecting millions of individuals worldwide with an estimated lifetime prevalence of 1-20% [1]. Ureteral stones are a frequent manifestation of urolithiasis and contribute to significant morbidity and healthcare burden [2]. Management options for ureteral stones range from conservative measures, such as observation and pain management, to invasive interventions, including surgical stone removal and ureteroscopic lithotripsy [3]. However, medical expulsive therapy (MET) has emerged as a widely accepted non-invasive treatment modality for facilitating spontaneous stone passage and relieving symptoms associated with ureteral stones [4].

MET typically involves the administration of pharmacological agents, such as alpha-blockers (e.g., tamsulosin) and nonsteroidal anti-inflammatory drugs (e.g., diclofenac), to promote ureteral relaxation, reduce ureteral edema, and relieve pain [5]. While MET is effective in facilitating stone expulsion in a significant proportion of patients, the success rates vary widely across [6]. Consequently, there is growing interest in identifying predictive factors that may help stratify patients according to their likelihood of successful stone expulsion with MET.

Recent research has focused on exploring the predictive role of radiological and hematological parameters in determining the success of MET for ureteral stones. Radiological parameters, including stone size, location, and degree of hydronephrosis, have been implicated as potential predictors of spontaneous stone passage [7]. Similarly, hematological markers of inflammation, such as C-reactive protein (CRP) levels and neutrophil-to-lymphocyte ratio (NLR), have been proposed as indicators of ureteral obstruction and inflammatory response, which may influence stone expulsion dynamics [8]. CRP and NLR are used as markers for predicting MET success due to their roles in reflecting the inflammatory status and systemic response to ureteral stones. High levels of these markers suggest increased inflammation, which might impair the effectiveness of MET and influence treatment planning. Despite the growing body of evidence supporting the predictive value of these parameters, comprehensive studies are needed to elucidate their role in predicting MET success and guiding clinical decision-making.

The primary objective of this study was to elucidate the relationship between radiological and hematological parameters and the success of MET for ureteral stone expulsion. Understanding the interplay between these parameters and treatment outcomes is essential to optimize patient selection and treatment planning in clinical practice. Additionally, this study aimed to explore secondary objectives, including the duration of ureteric stone expulsion, to provide further insights into the efficacy and timeline of MET.

## Materials and methods

The study was designed as a prospective observational cohort study conducted at the Department of Urology, tertiary care center, Patna, over 18 months. The cohort comprised consecutive patients who underwent MET for ureteric stones, adhering to the predefined inclusion and exclusion criteria. This study aimed to systematically observe and analyze the outcomes of MET in real-world clinical settings, providing valuable insights into the effectiveness and predictive factors associated with this therapeutic approach for ureteral calculi.

This study received ethical clearance from the Institutional Review Board (IRB) of Netaji Subhash Medical College and Hospital, with reference number CREC/2023/29. All procedures performed in this study involving human participants were conducted following the ethical standards outlined in the Declaration of Helsinki and its subsequent amendments. Informed consent was obtained from all the participants before their involvement in the study. Confidentiality of patient information was strictly maintained throughout the study, and data were anonymized to protect the privacy of participants.

Inclusion and exclusion criteria

The study included patients who met the specific criteria. The inclusion criteria were individuals aged between 18 and 50 years with a single ureteric stone measuring less than 10 mm. Conversely, exclusion criteria were defined to exclude certain conditions that might have confounded the study results. Patients with urinary tract infections (UTIs), proximal moderate-to-gross hydronephrosis (HDUN), solitary kidneys, or any associated diseases or anomalies of the genitourinary tract were excluded. Individuals diagnosed with chronic kidney disease (CKD) were excluded from the study. These criteria were established to ensure a homogeneous study population and minimize potential confounding factors that could affect the outcomes of MET for ureteral calculi. Moderate hydronephrosis is characterized by a noticeable enlargement of the renal pelvis and calyces while the kidney’s overall function remains relatively preserved. In contrast, gross hydronephrosis represents a more severe condition, with extensive swelling of the renal pelvis, calyces, and sometimes the entire kidney, leading to a significant reduction in renal function.

Patients who presented with symptoms suggestive of ureteric calculus underwent meticulous and thorough evaluation. This involved a systematic approach to gathering a comprehensive medical history, including details of any previous medical conditions, surgeries, medications, and specific symptoms related to the current presentation. Demographic data, such as age, sex, and pertinent personal information, were meticulously recorded to establish a robust baseline profile for the study cohort.

Following the assessment of medical history, a detailed clinical examination was conducted to evaluate vital signs, general appearance, and any specific physical signs associated with ureteric stone disease. Special attention was paid to symptoms such as flank pain, hematuria, or urinary symptoms, which are indicative of ureteral calculi.

Furthermore, to gain deeper insights into patients' overall health status and potential factors influencing the success of MET, a comprehensive array of hematological and biochemical investigations were carried out. These investigations encompassed a range of parameters, including hemoglobin (Hb) levels, total leukocyte count (TLC), platelet count, serum creatinine levels, CRP levels, neutrophil percentage, NLR, and platelet-to-lymphocyte ratio (PLR). Each parameter was meticulously analyzed to identify any abnormalities or trends that could impact the outcome of the MET.

**Figure 1:**
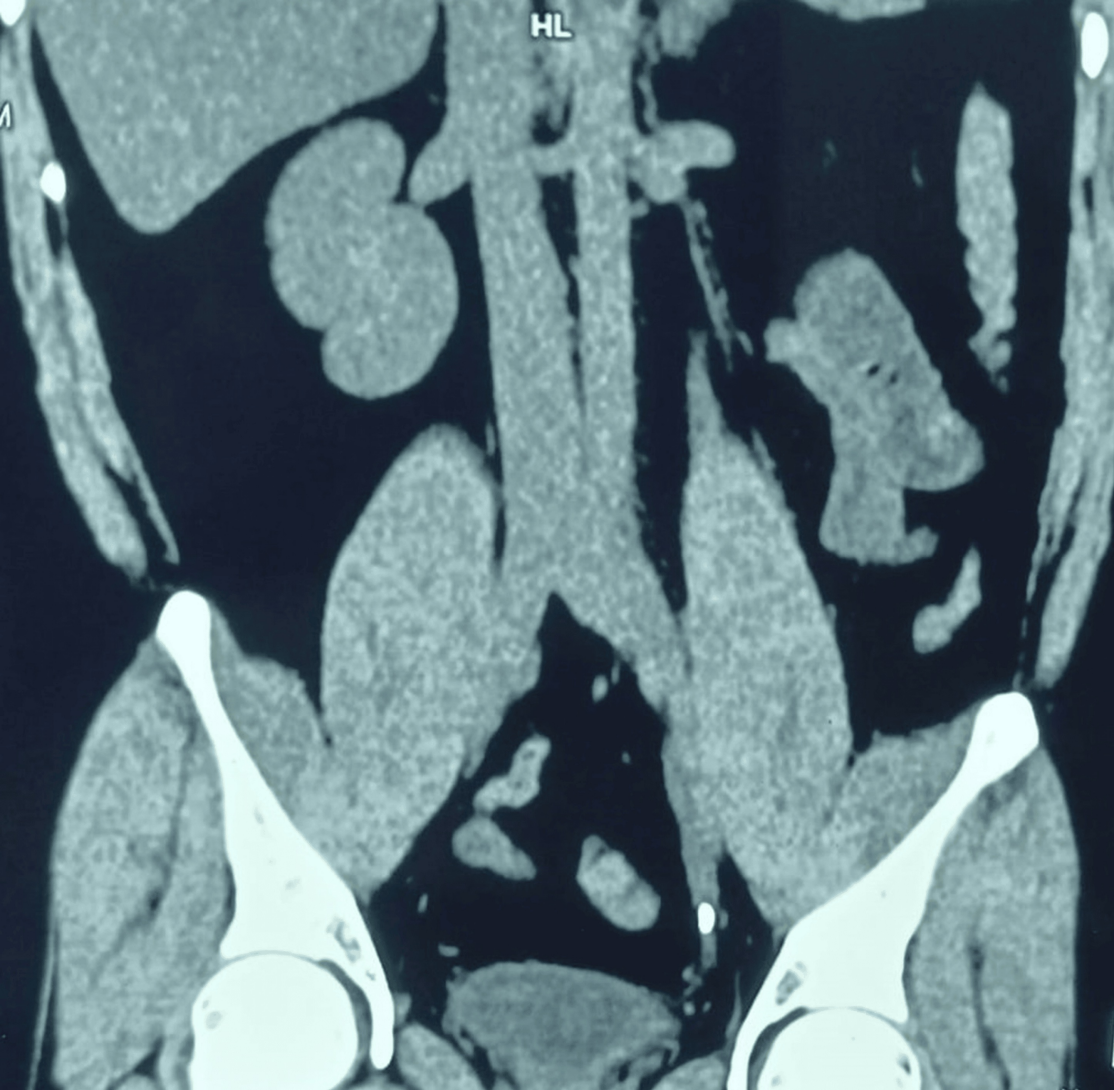
Non-contrast CT image showing 8.5x3.5 mm left distal ureteric calculus with proximal hydroureteronephrosis CT: computed tomography

In addition to clinical and laboratory assessments, radiological imaging was performed using computed tomography of the kidneys, ureters, and bladder (CT KUB) (Figure 1).

This imaging modality provided detailed visualization of the ureteric stone and allowed for the assessment of its characteristics, including longitudinal and transverse diameters, ureteric wall thickness, Hounsfield unit (HU) value, and the presence or absence of mild hydronephrosis. Upon completion of diagnostic evaluations, eligible patients were initiated on a MET regimen. This involved the administration of tamsulosin (0.4 mg) once daily for a duration of four weeks, along with diclofenac (50 mg) every eight hours as needed for pain relief for four weeks. The treatment was meticulously administered and monitored to ensure compliance and efficacy.

Following completion of the MET regimen for ureteral stone expulsion, the patients underwent a comprehensive follow-up evaluation. During the follow-up visit, a detailed review of the patient's medical history was conducted, with particular emphasis on assessing any changes in symptoms or signs of complications. Additionally, repeated clinical examinations were performed to monitor the patient's progress and response to treatment.

Furthermore, hematological parameters (Hb, TLC, and platelets) and kidney function tests (KFT) were reassessed to evaluate the patient's overall health status and response to treatment. A repeat CT KUB was also conducted to assess the status of stone expulsion and any changes in the stone characteristics.

Statistical analysis

The statistical analysis plan for this study commenced with data entry using an MS Excel spreadsheet (Microsoft Corporation, Redmond, Washington, United States). Statistical analyses were conducted using Statistical Package for the Social Sciences (SPSS) version 21.0 (IBM, Armonk, New York, United States). Categorical variables are presented as numbers and percentages (%), while continuous variables are expressed as mean ± standard deviation (SD) and median. The Kolmogorov-Smirnov test was used to assess normality, and nonparametric tests were employed if normality was rejected. The analysis involved using the unpaired t-test or Mann-Whitney test for quantitative variables if the datasets were not normally distributed, focusing on the success of MET for ureteral stone expulsion. Qualitative variables were analyzed using the chi-squared test or Fisher’s exact test. Additionally, univariate and multivariate logistic regression analyses were conducted to identify predictors of success of MET for ureteric stone expulsion. To explore correlations, Pearson correlation coefficient/Spearman rank correlation coefficients were computed between the quantitative variables and stone expulsion time. The significance level was set at p<0.05.

## Results

The demographic characteristics of the study participants revealed a balanced distribution across age groups and slight male predominance. Specifically, the majority of the participants were aged between 18 and 40 years with 90 (57.7%) being male and 66 (42.3%) females. These demographic features represented a representative sample for this study (Table 1).

**Table 1:** Demographic characteristics of study participants

Variable	Category	Frequency (n)	Percentage (%)
Age (years)	18-30	60	38.5
	31-40	50	32.1
	41-50	46	29.5
Sex	Male	90	57.7
	Female	66	42.3

Radiological parameters were significantly associated with the success of MET for ureteral stone expulsion. Notably, smaller transverse (p<0.001) and longitudinal (p<0.001) stone diameters, thinner ureteric walls (p<0.001), and lower HU values (p<0.001) were strongly correlated with higher MET success rates. Additionally, hydronephrosis was significantly associated with stone expulsion success (p<0.001) (Table 2).

**Table 2:** Radiological parameters and stone expulsion success P-value <0.05 is considered significant. MET: medical expulsive therapy; HU: Hounsfield unit

Parameter	Mean±SD (median)	Success in MET (mean±SD)	P-value
Transverse diameter of stone (mm)	7.8±1.5 (8.0)	80.2±10.3	<0.001
Longitudinal diameter of stone (mm)	9.2±2.0 (9.0)	79.5±12.1	<0.001
Ureteric wall thickness (mm)	2.5±0.8 (2.4)	75.3±9.8	<0.001
HU	900±150 (920)	78.9±11.5	<0.001
Presence of hydronephrosis	-	60.0%	-

Out of the total 156 patients who underwent MET for ureteral stones, the success rates varied across different parameters. Specifically, 120 patients (77%) achieved successful stone expulsion when considering the transverse diameter of the stone, 115 patients (74%) succeeded with the longitudinal diameter, 110 patients (71%) had successful outcomes related to ureteric wall thickness, and 125 patients (80%) experienced success in the context of HU measurements. Additionally, the presence of hydronephrosis was noted in 94 patients (60%) who experienced success in MET.

Hematological parameters were also significantly associated with MET success. Specifically, lower CRP levels (p=0.032) and PLR (p=0.076) were correlated with higher rates of stone expulsion success, while the white blood cell (WBC) count (p=0.214) and NLR (p=0.049) exhibited trends toward significance (Table 3).

**Table 3:** Hematological parameters and stone expulsion success P-value <0.05 is considered significant. MET: medical expulsive therapy; WBC: white blood cell; NLR: neutrophil-to-lymphocyte ratio; PLR: platelet-to-lymphocyte ratio; CRP: C-reactive protein

Parameter	Mean±SD (median)	Success in MET (mean±SD)	P-value
CRP (mg/L)	8.5±3.2 (8.0)	9.6±4.1	0.032
WBC count (x10^9/L)	9.2±2.1 (9.0)	9.5±2.3	0.214
NLR	2.1±0.8 (2.0)	2.4±0.9	0.049
PLR	145±30 (142)	150±35	0.076

Logistic regression analysis confirmed the predictive value of certain parameters for MET success. In the univariate analysis, a smaller transverse diameter of the stone (OR=0.65, p<0.001), lower levels of CRP (OR=1.28, p=0.002), and lower NLR (OR=1.52, p<0.001) were associated with increased odds of successful stone expulsion. After adjusting for confounding factors in multivariate analysis, these associations remained significant, highlighting their independent predictive value for MET success (Table 4).

**Table 4:** Univariate and multivariate logistic regression analysis for predictors of MET success P-value <0.05 is considered significant. CRP: C-reactive protein; NLR: neutrophil to lymphocyte ratio; MET: medical expulsive therapy

Predictor	Univariate odds ratio (95% CI)	P-value	Multivariate adjusted odds ratio (95% CI)	P-value
Transverse diameter of stone (mm)	0.65 (0.55-0.77)	<0.001	0.72 (0.60-0.85)	<0.001
CRP (mg/L)	1.28 (1.10-1.48)	0.002	1.14 (0.96-1.35)	0.124
NLR	1.52 (1.27-1.82)	<0.001	1.41 (1.16-1.72)	<0.001

Correlation analysis demonstrated significant relationships between the quantitative variables and stone expulsion time. Specifically, a negative correlation was observed between the transverse diameter of the stone (Pearson's r=-0.45, p<0.001) and stone expulsion time, indicating that larger stones take longer to pass through the ureter. Conversely, a positive correlation was noted between ureteric wall thickness (Pearson's r=0.30, p=0.012) and stone expulsion time, suggesting that thicker ureteric walls impede stone passage. Additionally, CRP levels were positively correlated with stone expulsion time (Pearson's r=0.25, p=0.035), indicating the potential role of inflammation in delaying stone passage. These findings provide valuable insights into the factors that influence stone expulsion dynamics during the MET (Table 5).

**Table 5:** Correlation between quantitative variables and stone expulsion time P-value <0.05 is considered significant. CRP: C-reactive protein

Variable	Pearson correlation coefficient	P-value	Spearman rank correlation coefficient	P-value
Transverse diameter of stone (mm)	-0.45	<0.001	-0.42	<0.001
Ureteric wall thickness (mm)	0.30	0.012	0.28	0.021
CRP (mg/L)	0.25	0.035	0.23	0.048

## Discussion

This study aimed to evaluate the predictive role of radiological and hematological parameters in the success of MET for ureteral stones. The findings revealed significant associations between various parameters and stone expulsion success, providing valuable insights into the potential predictors and mechanisms underlying the efficacy of MET.

Radiological parameters, including the transverse and longitudinal diameters of the stone, ureteric wall thickness, HU, and the presence of hydronephrosis, were found to be important predictors of MET success. Smaller stone dimensions, as reflected by reduced transverse and longitudinal diameters, were consistently associated with higher rates of stone expulsion success. This aligns with previous studies highlighting the impact of stone size on spontaneous stone passage [9,10]. The rationale behind this association lies in the concept that smaller stones have a higher probability of traversing the ureter owing to reduced mechanical obstruction and lower frictional resistance against the ureteric walls [10,11]. Additionally, thinner ureteric walls were correlated with increased stone expulsion success, suggesting that ureteric dilatation may facilitate stone passage [6,12]. Furthermore, lower HU values, indicative of lower stone density, were associated with higher MET success rates. Stones with lower density are often composed of less crystalline materials, making them more amenable to fragmentation and passage [13,14]. The presence of hydronephrosis has also emerged as a significant predictor of stone expulsion success, underscoring the role of urinary obstruction in influencing treatment outcomes [15].

In parallel, hematological parameters, including CRP levels, WBC, NLR, and PLR, were identified as potential biomarkers for predicting MET success. Lower CRP levels were associated with higher rates of stone expulsion success, suggesting that reduced systemic inflammation may facilitate stone passage [16]. This is supported by evidence indicating that inflammatory processes may contribute to ureteral edema and impaired peristalsis, thereby hindering stone expulsion [17]. Similarly, a lower NLR was predictive of MET success, indicating a more favorable systemic inflammatory profile [18]. Although not statistically significant, the PLR exhibited a trend toward association with stone expulsion success, warranting further investigation.

Logistic regression analyses confirmed the independent predictive value of these parameters for MET success even after adjusting for potential confounders. Specifically, smaller stone size, lower CRP level, and lower NLR were consistently associated with increased odds of successful stone expulsion. These findings underscore the importance of considering both radiological and hematological parameters in clinical decision-making regarding MET initiation and optimization.

Correlation analysis further elucidated the relationship between the quantitative variables and stone expulsion time. Larger stone dimensions were associated with prolonged stone expulsion time, highlighting the mechanical challenges posed by larger stones traversing the ureter. Conversely, thicker ureteric walls and higher CRP levels were correlated with longer stone expulsion times, reflecting potential physiological barriers to stone passage. These findings provide valuable insights into the multifactorial nature of stone expulsion dynamics during MET and emphasize the need for personalized treatment approaches based on individual patient characteristics [4].

The findings of this study have significant clinical implications for the management of patients undergoing MET for ureteral stones. First, the identification of specific radiological and hematological parameters as predictors of MET success provides clinicians with valuable tools for customizing treatment strategies based on individual patient characteristics. By integrating these predictive factors into clinical decision-making, healthcare providers can optimize treatment outcomes and minimize unnecessary interventions for patients with a higher likelihood of spontaneous stone passage. Furthermore, recognizing systemic inflammatory markers such as CRP levels and NLR as potential biomarkers for MET success highlights the importance of considering the systemic inflammatory response in stone management [19]. This insight may prompt further investigation into the role of anti-inflammatory agents or adjunctive therapies in enhancing stone expulsion rates and reducing treatment-related morbidity.

Looking ahead, these findings pave the way for future research endeavors aimed at refining predictive models for MET success and elucidating the mechanisms underlying treatment outcomes. Prospective studies involving larger multicenter cohorts are warranted to validate the findings of this study and establish robust predictive models incorporating a comprehensive range of clinical, radiological, and biochemical parameters. Longitudinal studies with extended follow-up periods are crucial for assessing the durability of treatment success and evaluating the risk of stone recurrence after MET. Additionally, translational research efforts focusing on the molecular pathways involved in stone formation, inflammation, and ureteral dynamics may uncover novel therapeutic targets to improve treatment efficacy and patient outcomes.

Integrating advanced imaging modalities, such as functional MRI and dynamic CT imaging, in clinical practice holds promise for providing valuable insights into ureteral motility and facilitating real-time monitoring of stone expulsion dynamics during MET. These technologies have the potential to enhance treatment planning and optimize patient outcomes by enabling clinicians to tailor interventions based on real-time anatomical and functional data [20]. Ultimately, the continued advancement of personalized medical approaches tailored to individual patient profiles offers considerable potential for optimizing the management of ureteral stones and improving patient quality of life. By leveraging multidisciplinary collaborations and emerging technologies, clinicians and researchers can work together to address the unmet needs of ureteral stone management and enhance the overall efficacy and safety of treatment approaches [21].

While this study provides valuable insights into the predictive role of radiological and hematological parameters in MET success, several limitations warrant consideration. First, the observational nature of the study precludes the establishment of causality, and further prospective randomized controlled trials are warranted to validate these findings. Additionally, the study was conducted at a single center, which may limit the generalizability of the results to a broader population. Moreover, studies focused on short-term outcomes of MET, and long-term follow-up data are necessary to assess the durability of treatment success and recurrence rates. Finally, the study did not account for potential confounding factors such as dietary habits, fluid intake, and medication adherence, which may influence stone expulsion dynamics.

## Conclusions

In conclusion, this study highlights the predictive value of radiological and hematological parameters for determining the success of MET for ureteral stones. A smaller stone size, lower CRP levels, and favorable inflammatory profiles were identified as potential predictors of MET success, providing valuable insights into personalized treatment strategies for patients with ureteral calculi. Future research endeavors should focus on validating these findings in larger multicenter cohorts and elucidating the underlying mechanisms that drive treatment outcomes.
